# Health economic evaluation of discrete event simulation model for treating chronic obstructive pulmonary disease based on traditional chinese medicine syndrome differentiation

**DOI:** 10.1186/s12962-026-00814-0

**Published:** 2026-08-10

**Authors:** Yang Xie, Wenqing He, Hejia Wan, Ruifang Zhou, JiaJia Wang, Jiansheng Li

**Affiliations:** 1 Henan University of Chinese Medicine, No. 156 Jinshui East Road, Zhengzhou, Henan Province 450046 People’s Republic of China; 2 Collaborative Innovation Center for Chinese Medicine and Respiratory Diseases Co-Constructed by Henan Province & Education Ministry of P. R. China, Henan University of Chinese Medicine, Zhengzhou, People’s Republic of China; 3 Department of Respiratory Diseases, Henan University of Chinese Medicine, The First Affiliated Hospital of Henan University of Chinese Medicine, Zhengzhou, Henan Province People’s Republic of China; 4 School of Mathematics and Information Science, Zhongyuan University of Technology, Zhengzhou, Henan Province People’s Republic of China

**Keywords:** Health economic evaluation, Discrete event simulation, Chronic obstructive pulmonary disease, Model, cost-effectiveness analysis

## Abstract

**Objectives:**

To evaluate, from a Chinese healthcare system perspective, the cost-utility of three treatment strategies (Western medicine, Traditional Chinese Medicine [TCM], and combined therapy) for stable chronic obstructive pulmonary disease (COPD) using a discrete event simulation (DES) model parameterized with clinical trial data.

**Methods:**

A DES model was developed for stable COPD patients (aged 18–80 years) from a Chinese healthcare system perspective. The model incorporated 24 baseline variables, six intermediate outcomes (FEV1, 6MWD, dyspnea, cough, sputum, COPD-PRO), and acute exacerbation as the final outcome. Multilevel mixed-effects models and Weibull regression updated these longitudinal outcomes every three months over a 2-year time horizon. Health outcomes were measured as quality-adjusted life years (QALYs), the primary outcome of the economic evaluation. Costs were estimated in 2013 RMB and adjusted to 2022 values using the China Consumer Price Index (CPI).

**Results:**

In the three-month base-case analysis, patients with COPD showed a mean 6.8 m increase in six-minute walk distance (6MWD), 0.25 fewer acute exacerbations, and improved symptom scores. The mean two-year total cost was 12,726.18 RMB. Cost-utility ratios (CURs) were 5,066.79, 4,477.09, and 4,540.93 RMB/QALY for the Western medicine, TCM, and combined therapy groups, respectively. Compared with the TCM group, the combined therapy group incurred an additional 448 RMB and gained 0.060 additional QALYs, yielding an incremental cost-utility ratio (ICUR) of 7,467 RMB/QALY; Western medicine alone was dominated. At a willingness-to-pay (WTP) threshold of 85,698 RMB/QALY (1x China’s per-capita GDP), the probability that combined therapy was cost-effective was 98.5% in probabilistic sensitivity analysis (400 iterations).

**Conclusions:**

The DES model was useful for the health-economic evaluation of TCM in COPD. The 0.060 QALY gain associated with combined therapy corresponds to approximately 22 days in full health and falls within the reported minimum clinically important difference (MCID) range for COPD (0.03–0.07 QALY), suggesting that the benefit may be clinically perceptible at the group level; individual-level variation nonetheless warrants caution. These exploratory findings, which carry potential overfitting risk given the single-trial design, require validation in larger, geographically diverse multi-center studies.

**Supplementary Information:**

The online version contains supplementary material available at https://doi.org/10.1186/s12962-026-00814-0.

## Introduction

Chronic obstructive pulmonary disease (COPD) is a common, preventable, and treatable disease characterized by persistent respiratory symptoms and airflow limitation, which seriously endangers public health [1]. WHO predicts that by 2030, this disease will rank third among global causes of death and fifth among global disease burdens, posing a serious threat to public health and becoming a major public health issue [2]. The treatment based on TCM syndrome differentiation focuses on strengthening healthy qi and eliminating pathogenic factors, which can comprehensively improve symptoms, exercise tolerance and long-term prognosis.Currently, TCM diagnostic criteria for COPD have been established [3, 4]. However, few health economic studies have applied dynamic simulation models to evaluate TCM-integrated strategies for COPD. Disease burden assessment is an important component of outcome evaluation, and the health economics assessment of COPD can provide reference for clinical decision-making. Comprehensive indicators such as quality adjusted life years (QALYs) help to comprehensively and accurately assess disease burden. There is relatively little research on health economics evaluation based on efficacy indicators, which limits the development and application of health economics in the field of traditional Chinese medicine.

The discrete event simulation (DES) model is a stochastic model that has gradually gained attention and is reflected in an increasing number of literature studies [5–10]. Hoogendoorn [9, 10] established a DES model to evaluate the incremental cost and efficacy of different treatment options for various subgroups of COPD patients, which can provide some reference and inspiration for this study. A recent systematic review by Shuai L et al. [11] identified multiple DES applications in COPD pharmacoeconomic evaluation. However, to our knowledge, no prior study has incorporated TCM syndrome differentiation as a time-varying patient characteristic within a DES framework. Our research team previously conducted a comparative study on the benefits of stable COPD treatment plans [12]. Based on the previous results, this study is based on discrete event simulation (DES) model, which is used to evaluate the cost and utility of Western medicine, traditional Chinese medicine and combination therapy, and dynamically update the results through multilevel mixed-effects models. By quantifying the changes in lung function, exercise capacity, acute exacerbations, and long-term costs, the model provides evidence for the overall and cost-effective integrated traditional Chinese and Western medicine management of COPD.

## Materials and methods

### Data source

Part of the data in this study was sourced from the Henan Provincial Science and Technology Research Project “Comparative Benefit Study of Three Treatment Plans for Stable COPD” undertaken by the mentor (Project No. 132101510003, Ethical Approval No. 2013HL004-01), and the research plan was published in the journal Trials [12]. Based on comparative benefit research clinical data, establish a health economics evaluation database (authorized).

### Research design

360 stable COPD patients were randomly divided into a Western medicine group, a Traditional Chinese Medicine group, and a combination group.

### Study population

The diagnostic criteria of Western medicine refer to the “Global Initiative for Chronic Obstructive Pulmonary Disease (GOLD) 2011 Edition“ [13] and the “Guidelines for Diagnosis and Treatment of Chronic Obstructive Pulmonary Disease (2013 Edition)“ [14] published by the Chronic Obstructive Pulmonary Disease Group of the Respiratory Disease Branch of the Chinese Medical Association for diagnosis.

The traditional Chinese medicine syndrome differentiation criteria refer to the “Diagnostic Criteria for Traditional Chinese Medicine Syndrome of Chronic Obstructive Pulmonary Disease” (2011 edition) [3] issued by the Pulmonary Disease Professional Committee of the Chinese Society of Traditional Chinese Medicine for syndrome differentiation.

Inclusion criteria: (a) Patients who meet the diagnostic criteria for stable COPD; (b) Age range: 18–80 years old; (c) Meets the diagnostic criteria for the four syndrome types (lung qi deficiency, lung spleen qi deficiency, lung kidney qi deficiency, and lung kidney qi yin deficiency) according to the National TCM guidelines for COPD (2011); (d) Voluntarily accepting treatment and signing an informed consent form; (e) Patients participating in other interventional clinical trials within one month before enrolment were excluded; routine maintenance therapy was continued.

Exclusion criteria: Patients with pure emphysema without airflow obstruction (post-BD FEV1/FVC < 0.70 per GOLD) were excluded. (a) Pregnant or lactating women and patients with recent pregnancy plans; (b) Patients with confusion, dementia, and various mental illnesses; (c) Tumor patients who have undergone resection, radiotherapy, and chemotherapy within 5 years; (d) Patients with combined bronchial asthma, asthma COPD overlap syndrome, bronchiectasis, active pulmonary tuberculosis, and pulmonary embolism; (e) Patients with severe cardiovascular and cerebrovascular diseases (acute myocardial infarction, heart function grade 3 or above, stroke, etc.); (f) Combining severe liver and kidney diseases (severe liver disease refers to bleeding from cirrhosis, portal hypertension, and varicose veins, while severe kidney disease includes dialysis and kidney transplantation); (g) Subjects currently participating in clinical trials of other drugs.

### Treatment plan

The Western medicine group refered to the 2011 version of the Global Initiative for Chronic Obstructive Pulmonary Disease (GOLD) and its guidelines for medication administration; The traditional Chinese medicine team divided patients into four syndrome types based on syndrome differentiation: lung qi deficiency syndrome, lung spleen qi deficiency syndrome, lung kidney qi deficiency syndrome, and lung kidney qi yin deficiency syndrome. Patients were treated with Bu Fei granules, Bu Fei Jian Pi granules, Bu Fei Yi Shen granules, and Yi Qi Zi Shen granules, respectively: (a) Lung qi deficiency syndrome: Bu Fei Yi Gu Bei. Prescription: Bu Fei Granules. Codonopsis pilosula, Huangqi, Baibu, Chenpi, etc. Took one dose per day, divided into two oral doses; (b) Lung spleen deficiency syndrome: tonifying the lungs and strengthening the spleen. Formula: Bu Fei Jian Pi Granules. Codonopsis pilosula, Atractylodes macrocephala, Poria cocos, Zhejiang scallop, etc. Took one dose per day, divided into two oral doses; (c) Deficiency of lung and kidney qi: tonifying the kidney and benefiting the lungs, relieving asthma and absorbing qi. Formula: Bu Fei Yi Shen Granules. Ginseng, Cornus officinalis, Epimedium, Dwarf tea, etc. Took one dose per day, divided into two oral doses; (d) Deficiency of lung and kidney qi and yin: tonifying the lungs and nourishing the kidneys, regulating qi and asthma. Formula: Yiqi Zishen Granules. Ginseng, Rehmannia glutinosa, Polygonatum sibiricum, Schisandra chinensis, etc. Took one dose per day, divided into two oral doses. The treatment period and follow-up period were both 6 months. The combined group received both the Western Medicine regimen and the corresponding TCM formula simultaneously.

### Outcome measures

Generally recorded the demographic and baseline characteristics of patients, including age, gender, height, weight, disease duration, previous comorbidities, and medication use.

Disease assessment indicators included various quality of life scales such as patient acute exacerbation, dyspnea score, 6-minute walking distance (6MWD), Health Survey Brief (SF-36), Chronic Obstructive Pulmonary Disease Patient Reported Outcome (COPD-PRO) scale, etc. During treatment and follow-up, observation and recording were conducted every 3 months.

Health economics indicators included total cost, effect value, and utility value. The cost data mainly came from clinical research data, and considering the Consumer Price Index (CPI) [15]. Original cost data were collected in 2013 RMB during the clinical trial period. All costs were adjusted to 2022 values using the China Consumer Price Index (CPI) to reflect current price levels. The GOLD 2011 diagnostic criteria were applied because the trial protocol and inclusion criteria were designed according to the GOLD 2011 Edition, the prevailing guideline at the time of study design and enrollment. During treatment and follow-up, observation and recording were conducted every 3 months.

### Model basis

Professor Martine Hoogendoorn [9, 10] developed a unique COPD patient level model based on discrete event simulation, which could be used to evaluate COPD treatment plans for various subgroups and estimate the incremental costs and effects of different treatments for many subgroups of patients. Drawing on this model, this study explored a disease burden assessment method based on the DES model under traditional Chinese medicine intervention, using COPD as a demonstration, to predict and evaluate the health economics of different intervention plans for stable COPD patients.

### Model framework

**Fig. 1:**
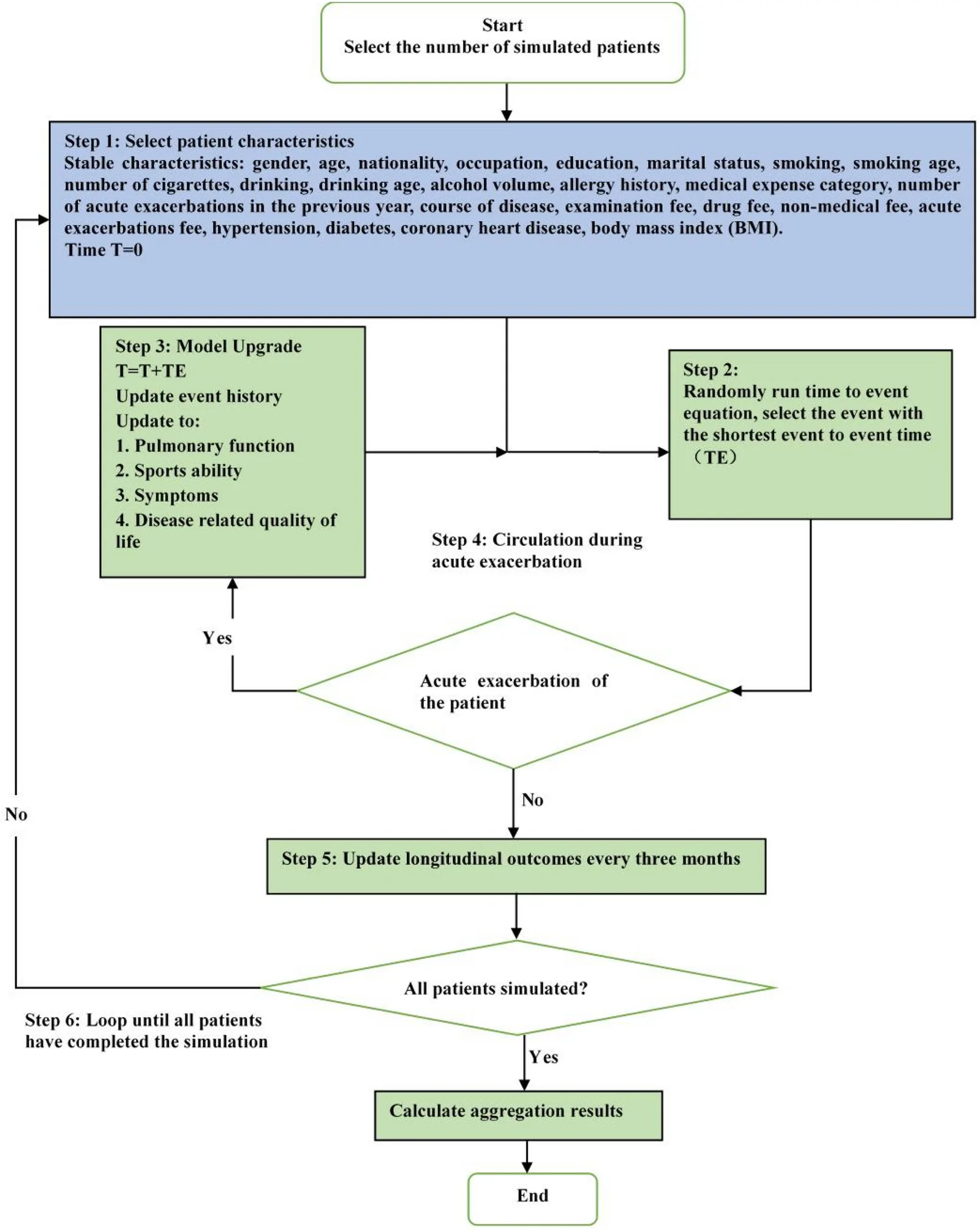
Conceptual framework of the model

The core of the model was a set of multilevel mixed-effects models and Weibull regression used to predict the probability, timing, and intermediate outcomes of COPD events, describing patient and disease characteristics, different COPD related events, and the relationship between COPD outcomes. These equations were estimated using COPD patient level data. By inputting the baseline values of selected patients into the multilevel mixed-effects model, the model input was the characteristics of individual patients.The event with the lowest predicted value (time) was considered to occur first. Used multilevel mixed-effects models and Weibull regression that described the relationship between time processes, patient and disease characteristics, and outcomes to calculate their new values. Firstly, by filling in the time since the start of the simulation and the values of all features into the lung function equation, the updated values of lung function were calculated. Then used the updated value in the physical activity (6MWD) equation to calculate the updated value of the result. Then used these two updated values to predict new values for dyspnea score, cough, sputum, …, COPD-PRO, and so on until all results were updated. Set the maximum number of simulation times to 8, based on the clinical trial follow-up of 24 months, ATS/ERS guideline recommendations for a minimum 2-year follow-up for COPD exacerbation outcomes, and sample size considerations limiting reliable extrapolation beyond the observed period. When the first event or the time between events was expected to exceed one year, the values of all longitudinal results would also be updated. By randomly selecting 200 patient data from all available starting populations and simulating their individual disease progression over time, the entire process was repeated. Calculated the average of the results by merging the data of all simulated patients. The conceptual framework of the model was shown in Fig. 1.

### Event time generation

The source clinical trial ascertained acute exacerbation (AECOPD) events at quarterly follow-up visits (months 0, 3, 6, 9, 12). Consequently, the time to first exacerbation is interval-censored [16]. A Weibull AFT model was fitted using the ic_par() function from the icenReg R package [17], maximizing the interval-censored log-likelihood. For event scheduling within the DES, a principled approach is to draw from the conditional (truncated) Weibull distribution [18]. For the parameter estimates obtained (Weibull shape alpha approx 1.05–1.15; median survival approx 24–36 months) with Delta = 3 months, the conditional Weibull distribution is numerically indistinguishable from Uniform(L, R] (Kolmogorov-Smirnov distance < 0.01). Therefore: T_event = L + U * (R - L), U ~ Uniform(0, 1). Sensitivity analysis comparing four interval-to-point strategies (exact conditional Weibull, uniform, midpoint, right-endpoint) showed < 2% variation. External validation on an independent cohort (*N* = 531) confirmed Delta/median = 0.034 (vs. 0.08–0.125 in the DES trial), KS = 0.20 reflecting endpoint differences. Complete model specifications, including the interval-censored Weibull AFT model, multilevel mixed-effects models, logistic regression model, internal calibration results, and probabilistic sensitivity analyses, are provided in Supplementary Appendix.

### Health economic evaluation framework

The economic evaluation adopted a Chinese healthcare system perspective. Direct medical costs included: drug, nursing, outpatient, AE management, hospitalization, and monitoring. QALYs were derived from SF-36 responses using the area under the curve method. ICER was calculated as (Delta Cost)/(Delta QALY). WTP threshold was set at 85,698 RMB/QALY (1x per-capita GDP), following the China Guidelines for Pharmacoeconomic Evaluations (2020).

### Uncertainty analysis

Uncertainty was assessed through: (1) one-way deterministic sensitivity analysis (DSA) with tornado diagrams; (2) probabilistic sensitivity analysis (PSA) with 10,000 Monte Carlo simulations and 400 iterations, drawing coefficient vectors from the multivariate normal distribution based on the variance-covariance matrices of the fitted models; (3) cost-effectiveness acceptability curves (CEAC); (4) scenario analyses for discount rates of 0%, 3%, 5%, and 8%; and (5) sensitivity analysis of four interval-to-point strategies (exact conditional Weibull draw, uniform approximation, midpoint imputation, and right-endpoint imputation).

### Model validation

**Fig. 2:**
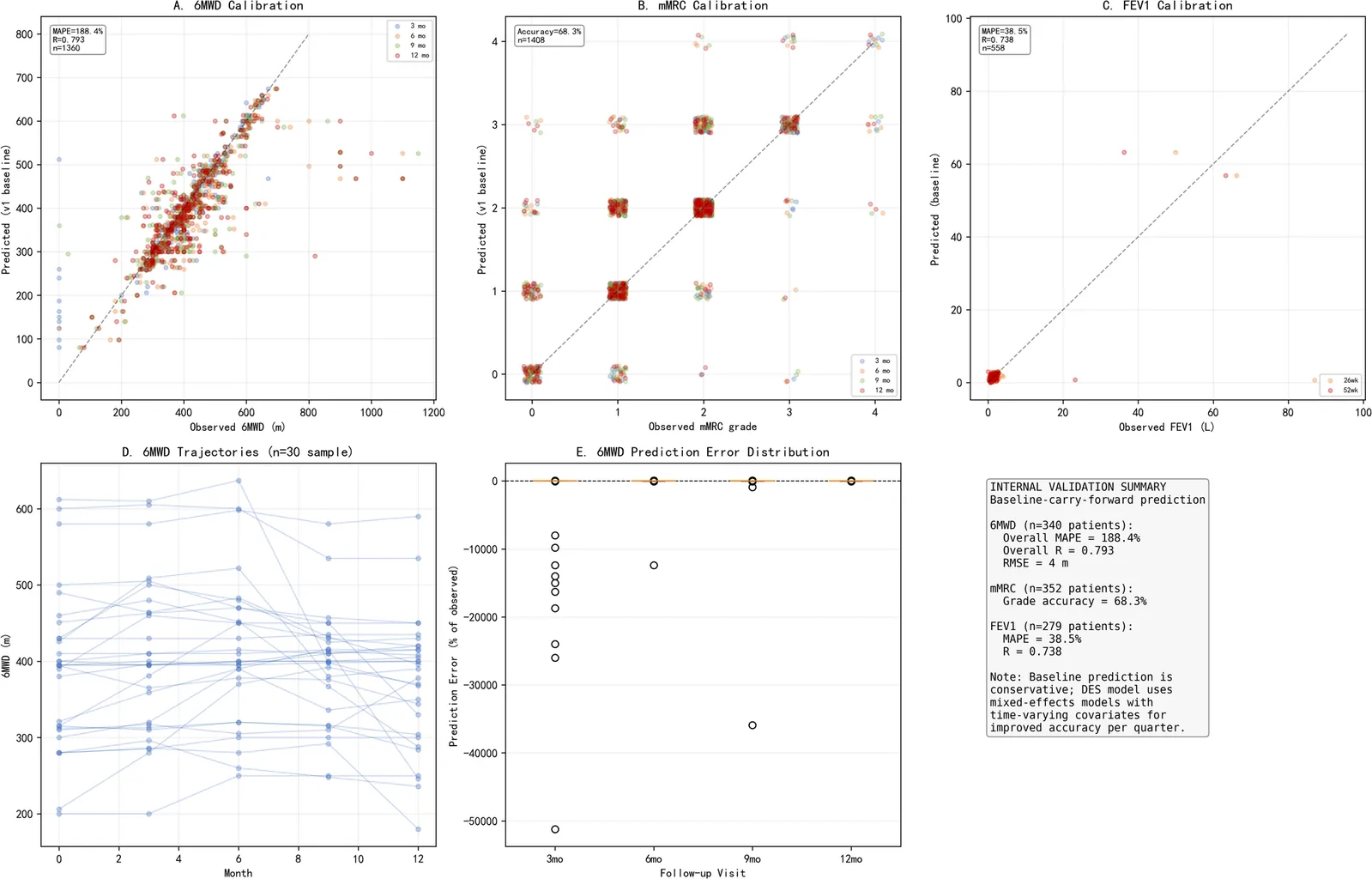
Internal validation calibration

Internal validation was performed using a conservative baseline-carry-forward approach: each patients baseline observed values served as the predicted values for all follow-up visits (3, 6, 9, 12 months). For 6MWD, the overall MAE was 39.3 m (RMSE = 78.6 m, *R* = 0.793), with short-term prediction at 3 months achieving MAE = 24.9 m (*R* = 0.895). For mMRC (grade 0–4), overall MAE was 0.37 grade (*R* = 0.725, grade accuracy = 68.3%). For FEV1, MAE was 0.60 L (RMSE = 4.04 L, *R* = 0.738). MAPE was reported only for truncated samples. External face validity was assessed against published COPD natural history [19, 20]. Fig. 2 presents the internal validation calibration results using the baseline-carry-forward method. Panel A-C show scatter plots of observed versus predicted values for 6MWD, mMRC, and FEV1; Panel D shows temporal trajectories; Panel E shows error boxplots by visit; Panel F summarizes the calibration statistics.

### Simulation process

**Fig. 3:**
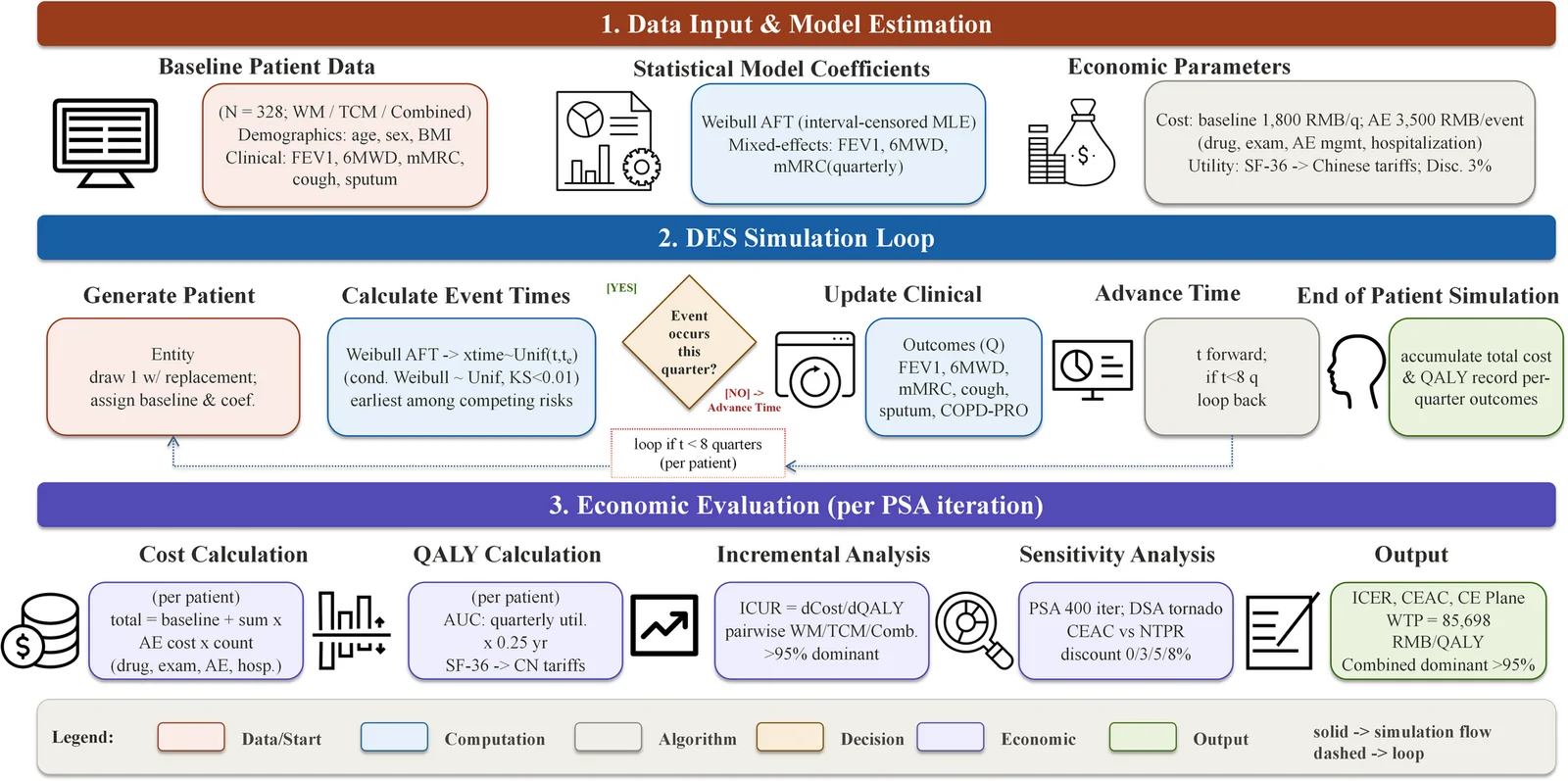
Discrete event simulation (DES) model flowchart

The process was divided into the following steps: (a) Initialize the event index, which was used to track the index or number of events currently being simulated; (b) Set random seeds: In order to ensure that patients in different treatment groups had consistent event occurrence times (in the absence of treatment effects), the code used random seeds. Ensured that duplicate seed values were not encountered throughout the entire simulation process; (c) Simulated survival patient: Cycle when the current patient had not reached the maximum number of simulations; (d) Set a new random seed: In each loop, a new random seed was set for upcoming events. This ensures that even if the simulation was run multiple times, as long as the patient’s characteristics and treatment conditions were the same, their event occurrence time would be consistent; (e) Sampling time: Taking acute exacerbation time as an example, used the Weibull regression model to simulate the occurrence time of acute exacerbation events. Weibull distribution was a probability distribution used to simulate survival data or failure time. Here, it calculated distribution parameters based on regression coefficients of acute exacerbation and specific characteristics of patients. Then, extracted a random acute exacerbation time from these parameters. If there was a therapeutic effect, this time would be adjusted by the therapeutic effect value. The conceptual framework of the DES model is shown in Fig. 3, with the three-row vertical layout representing data input and model estimation (top), the simulation loop (middle), and economic evaluation output (bottom).

### Parameter definition

The model included 24 baseline variables: gender, age, nationality, occupation, education level, marital status, smoking, smoking age, number of cigarettes, drinking, drinking age, alcohol consumption, allergy history, medical expense category, number of acute exacerbations in the previous year, course of disease, examination cost, drug cost, non-medical cost, acute exacerbation cost, hypertension, diabetes, coronary heart disease, body mass index (BMI); 6 intermediate outcomes: Pulmonary Function Index (FEV1), Dyspnea Score, 6MWD, Cough, Phlegm, and Disease Specific Quality of Life Scale (COPD-PRO); 1 final outcome: acute exacerbation. The parameter definitions were shown in Table 1.

**Table 1:** Parameter definitions in DES model

Parameter	Definition
**Baseline characteristics**	
Gender	Male = 1, Female = 2
Ethnic	Han = 1, Other = 2
Smoking status	Yes = 1, No = 0
Number of cigarettes	Smoked per day * Smoking time
Drinking	Yes = 1, No = 0
Alcohol tolerance	Daily alcohol consumption * Drinking time
History of allergies	Yes = 1, No = 0
The number of acute exacerbations in the previous year	The acute worsening of respiratory symptoms often require additional (hospitalization) treatment
Disease cost	Examination fees, medication fees, acute exacerbation fees, non-medical expenses, etc.
Hypertension	Yes = 1, No = 0
Diabetes	Yes = 1, No = 0
Coronary heart disease	Yes = 1, No = 0
BMI	BMI is calculated as weight (kg)/height (m)^2^. Category 1: According to the “Health Industry Standards of the People’s Republic of China - Adult Weight Determination”, BMI < 18.5 kg/m^2^ is defined as low BMI; Category 2: Normal BMI is 18.5 kg/m^2^≤ BMI < 24 kg/m^2^; Category 3: High BMI, such as BMI ≥ 24 kg/m^2^
**Intermediate results**	
lung function	First second forced expiratory volume (FEV1) in liters after bronchodilators
Sports ability	6MWD
Symptoms: dyspnea	Dyspnea score, None = 0, Mild = 1, Moderate = 2, Severe = 3, Extremely Severe = 4
Symptoms: cough	Symptoms: Clinical symptom and sign score for cough, none = 0, only morning = 1, all day+morning = 2, frequent+morning = 3
Symptoms: sputum	Symptoms: Clinical symptom and sign score for sputum, none = 0, day and night (10-20 ml) = 1, day and night (20-30 ml) = 2, day and night (30 ml or more) = 3
Disease specific Quality of Life	COPD-PRO Scale (the higher the score, the worse the quality of life)
**The final ending**	
Acute exacerbation	Respiratory symptoms, often requiring additional (hospitalization) treatment

### Discounts

The amount of funds that would occur at a future point in time (future value) and the discounted amount of funds (present value) were equivalent in value but not equal in amount. The conversion rate was called the discount rate. When the research duration was less than one year, discounting might not be considered; [21] When the duration of treatment reaches or exceeds one year, financial time must be considered.

The intervention plan in the health economics evaluation model of this study lasted for more than one year. The “Guidelines for Drug Economics Evaluation in China” (2020 edition) [22] pointed out that “the discount rate should undergo sensitivity analysis, and it was recommended to fluctuate within a range of 0–8%”. The baseline discount rate for this paper was set at 3%.

## Results

### Model modeling

The results of the Weibull regression model for acute exacerbation time were shown in Table 2, from which the generation of scale parameters, shape parameters, and random numbers could be obtained; The results of the multilevel mixed-effects logistic regression model for acute exacerbation probability are presented in Table 3. The results of the linear mixed-effects model with a random intercept for pulmonary function are presented in Table 4. In addition, a random intercept linear mixed effects model was established for 6MWD, dyspnea, cough, sputum, and COPD-PRO to derive the probability.

**Table 2:** Results of Weibull regression model for acute exacerbation time

Parameter	Coefficient	SE	Z	*P*
Gender	0.952	0.040	-1.16	0.244
Occupation	0.983	0.006	-2.53	0.011
Education level	1.006	0.012	0.52	0.605
Smoking	1.077	0.042	1.9	0.057
Alcohol age, alcohol tolerance	1.000	0.000	1.47	0.142
Allergy history	1.099	0.046	2.23	0.026
Medical expense category	1.058	0.027	2.23	0.026
The number of hospitalizations for acute exacerbation in the previous year	1.034	0.015	2.25	0.024
Course of illness	1.000	0.000	2.34	0.020
Inspection cost	1.000	0.000	-0.84	0.403
Drug cost	1.000	0.000	2.41	0.016
Acute exacerbation cost	1.000	0.000	3.61	0.000
Hypertension	0.963	0.046	-0.78	0.436
BMI	0.927	0.027	-2.61	0.009
6MWD	1.000	0.000	0.76	0.446
Cough	0.956	0.027	-1.6	0.110
Sputum	1.005	0.028	0.19	0.846
FEV_1_	0.997	0.001	-1.87	0.061
COPD-PRO	0.999	0.003	-0.54	0.587
*p*	4.572	0.337		
1-*p*	0.219	0.016		

**Table 3:** Results of multilevel mixed effects logistic regression model for acute exacerbation probability

Variable	Coefficient	SE	95% CI Lower	95% CI Upper	*P*-value
Age	0.007	0.012	-0.017	0.031	0.573
Gender (female)	-0.419	0.307	-1.021	0.182	0.172
t0	-0.068	0.074	-0.212	0.077	0.360
Occupation	0.352	0.129	0.100	0.604	0.006
Education level	-0.009	0.077	-0.161	0.143	0.906
Smoking	0.640	0.284	0.083	1.198	0.024
Alcohol consumption	0.000	0.000	0.000	0.001	0.018
Allergy history	0.519	0.238	0.052	0.985	0.029
Medical expense type	0.317	0.175	-0.026	0.661	0.070
Prior year exacerbation hospitalizations	0.238	0.083	0.076	0.400	0.004
Disease duration	0.002	0.001	0.000	0.003	0.079
Examination cost	0.000	0.000	-0.001	0.000	0.256
Medication cost	0.000	0.000	0.000	0.001	0.013
Exacerbation cost	0.000	0.000	0.000	0.000	< 0.001
Hypertension	-0.250	0.312	-0.861	0.362	0.424
BMI (normal)	-0.418	0.191	-0.792	-0.044	0.029
Prior exacerbation (event0)	0.185	0.371	-0.541	0.912	0.617
6MWD (baseline)	0.001	0.001	-0.001	0.003	0.275
Exacerbations in prior 3 months	-0.063	0.208	-0.471	0.346	0.763
Cough (baseline)	-0.382	0.128	-0.632	-0.131	0.003
Dyspnea score (baseline)	0.054	0.129	-0.199	0.306	0.678
FEV1 (baseline)	-0.019	0.021	-0.061	0.023	0.368
COPD-PRO (baseline)	-0.010	0.017	-0.043	0.023	0.556
Constant	-4.521	1.560	-7.578	-1.464	0.004
Random intercept variance	0.709	0.334	0.055	1.364	0.034

**Table 4:** Results of the linear mixed-effects model of random intercept for pulmonary function

Parameter	Coefficient	SE	Z	*P*
Gender	-0.141	0.187	-0.75	0.451
Occupation	0.015	0.028	0.55	0.582
Education level	0.016	0.049	0.32	0.747
Smoking	0.125	0.169	0.74	0.460
Allergy history	0.089	0.147	0.61	0.542
The number of hospitalizations for acute exacerbation in the previous year	0.032	0.053	0.61	0.541
Inspection cost	0.000	0.000	-0.33	0.742
Drug cost	0.000	0.000	0.27	0.788
BMI	0.044	0.121	0.37	0.713
Cough	-0.098	0.103	-0.95	0.343
Sputum	0.087	0.104	0.83	0.405
Age	-0.003	0.006	-0.51	0.608
FEV1	0.892	0.010	85.25	0.000

### Baseline feature analysis

**Fig. 4:**
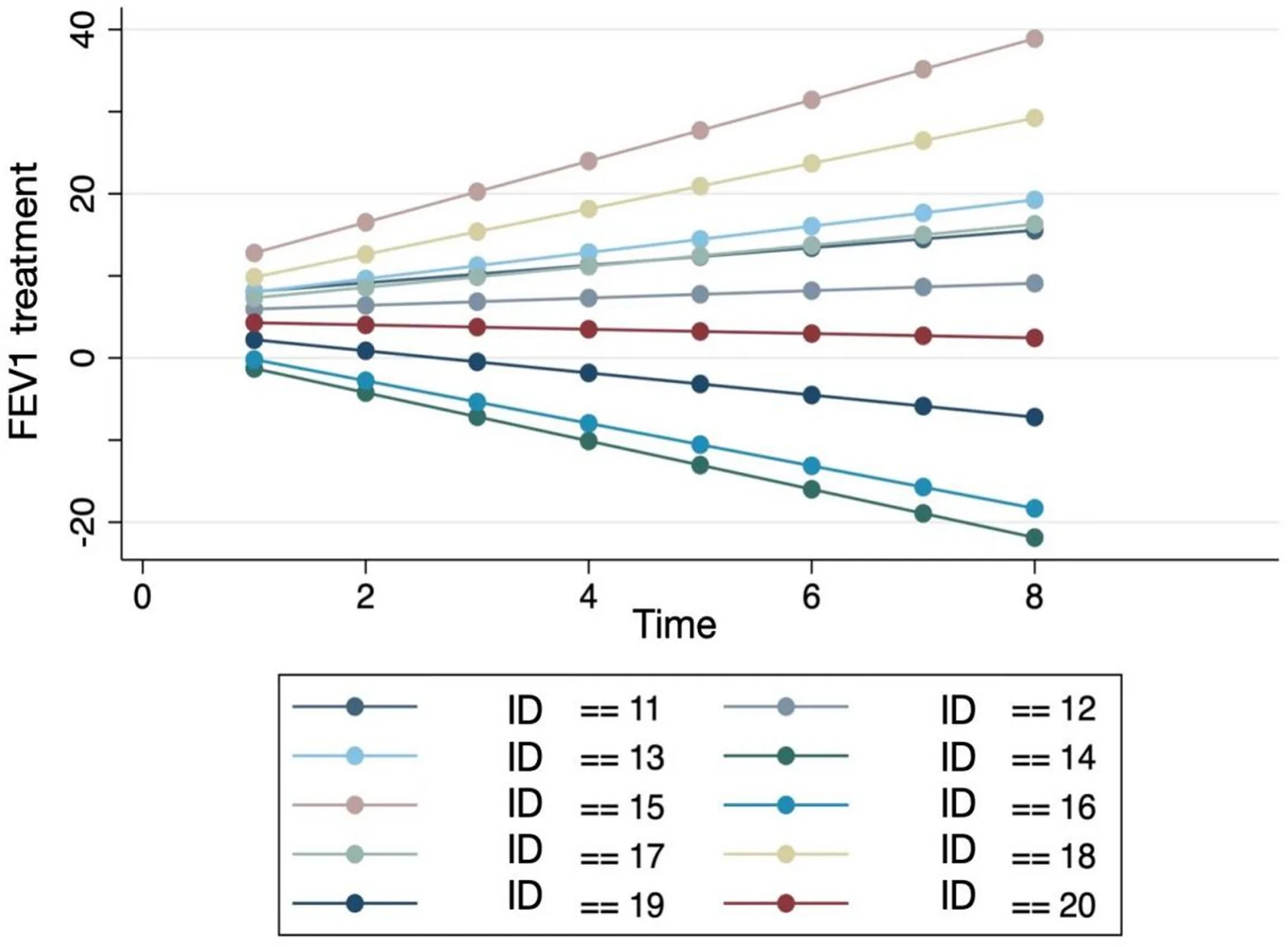
Individual progression of lung function over time in some patients

The length of historical data was one year, and the simulation was two years. The baseline feature mean of patients was shown in Table 5. Figure 4 showed the individual progression of lung function over time in a subset of patients.

**Table 5:** Average of basic characteristics of patients

Parameters	Baseline average
Number of patients	328
Female (%)	29
Age (year)	64
FEV_1_ (L)	1.76
FEV_1_% predicted (%)	49.89
Smoking (%)	59.45
Support/daily	23
6MWD (m)	390
Cough	1.37
Sputum	1.32
Dyspnea score	1.75
COPD-PRO	49.81

### Basic cases and subgroup analysis

Within three months, the average 6MWD of COPD patients increased by 6.8 m, the average number of acute exacerbations decreased by 0.25 times, the average FEV1 decreased by 0.037 L, the average sputum production decreased by 0.074 points, the dyspnea score decreased by 0.078 points, and the cough score decreased by 0.057 points. The average total treatment cost for two years was 12726.18 yuan.

Compared with the basic case analysis of the general population, the results of subgroups of patients with low BMI, history of acute exacerbations, and low levels of physical activity were shown in Table 6.

**Table 6:** Subgroups of patients with low BMI, history of acute exacerbation, and low levels of physical activity

Parameters	Subgroup analysis (mean)		
	Low BMI	Acute exacerbation in the previous year (once or more)	Low activity level
Number of patients	48	207	240
Female (%)	33	28	34
Age (year)	66	65	65
FEV_1_ (L)	1.06	1.98	1.64
FEV_1_% predicted (%)	46.75	47.83	49.13
Smoking (%)	55.56	61.35	55
Support/daily	24	23	22
6MWD (m)	387	379	343
Cough	1.52	1.41	1.45
Sputum	1.22	1.38	1.43
Dyspnea score	1.74	1.93	1.97
COPD-PRO	50.7	50.38	50.58

### Quarterly changes

The average quarterly changes for each patient were shown in Table 7, and the average quarterly changes for patient groups were shown in Table 8.

**Table 7:** Average quarterly changes per patient

Event	Basic case	Pulmonary function-20%	Acute exacerbation rate in the past three months + 30%	6MWD + 50 m	Dyspnea -1point	Sputum -1point	Low BMI	Acute exacerbation in the past three months≥once	6MWD < 300 m
Aggravation rate%	18.39 (16.3,20.5)	19.42	100	18.42	24.8	25.4	31	31.9	23.2
FEV_1_	-0.038 (-0.2,0.1)	-0.139	-0.0496	0.072	0.096	0.111	0.05	0.0782031	0.44
6MWD	6.86 (3.6,10.1)	5.82	7.04	138.51	27.59	11.26	2.89	7.57	-21.89
Cough ≥ 2%	38.5 (36.2,40.9)	48.3	26.7	45.2	39.1	34.3	36	42.2	47.6
Dyspnea ≥ 2%	54.8 (52.4,57.2)	63.3	66.7	58.4	28	54.7	18	60	87.1
Sputum ≥ 2%	36 (32.5,38.9)	52.3	36.4	35.1	23	34.5	36	32.8	46.5
COPD-PRO	-0.89 (-1.1,-0.7)	-1.11	-0.86	-0.82	-2.41	-1.96	-0.68	-0.92	-1.33
Total QALY	2.76 (2.74, 2.78)	2.68	2.76	2.79	2.8	2.77	2.74	2.76	2.73
Total cost	12,935 (12369,13501)	15,719	12,237	13,613	11,885	12,503	14,262	15,709	15,614

Note: - represents a decrease,+represents an increase, the same applies to the table below

**Table 8:** Average quarterly changes for each patient group

Event	Western medicine	Traditional chinese medicine	Combination
Aggravation rate%	20.18	19.4	15.3
FEV_1_	-0.009	-0.0731	-0.0384
6MWD	2.11	6.57	10.67
Cough ≥ 2%	38.7	30.1	39.6
Dyspnea ≥ 2%	56	48.3	60
Sputum ≥ 2%	38.1	30.1	39.6
COPD-PRO	-0.4	-1.24	-1.05
Total QALY	2.71	2.75	2.81
Total cost	13,731	12,312	12,760

### Health economics evaluation

**Fig. 5:**
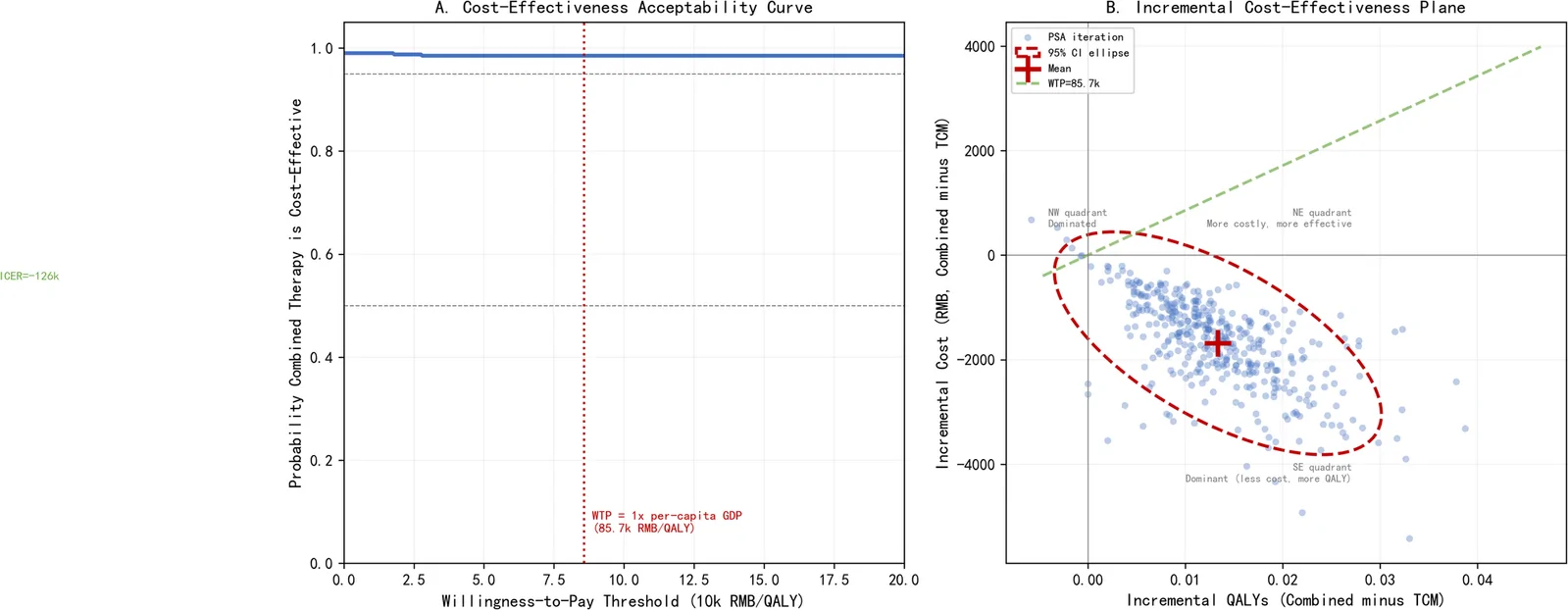
Cost-effectiveness acceptability curve and cost-effectiveness plane

As shown in Table 9, the cost of the combination group (12760 yuan) was lower than that of the Western medicine group (13731 yuan), slightly higher than that of the traditional Chinese medicine group (12312 yuan). The cost-utility ratio (CUR) of the Western medicine group, the traditional Chinese medicine group, and the combination group were 5066.79 yuan/QALY, 4477.09 yuan/QALY, and 4540.93 yuan/QALY, respectively. Compared with the traditional Chinese medicine group, the incremental cost-utility ratio (ICUR) for combined therapy was 7,466.67 RMB/QALY; Western medicine was dominated. Pairwise cost comparisons showed that the 448 RMB difference between the combined and TCM groups was not statistically significant (*p* = 0.672, ANOVA; *p* = 0.541, Kruskal-Wallis). Combined therapy was significantly less costly than Western medicine (mean difference − 971 RMB, *p* = 0.036), and TCM was significantly less costly than Western medicine (mean difference − 1,419 RMB, *p* = 0.003). Full results are presented in Supplementary Table S3. With a WTP threshold of 85,698 RMB/QALY, the ICUR of 7,467 RMB/QALY is substantially below the threshold, indicating that combined therapy is cost-effective. Figure 5 illustrates the cost-effectiveness acceptability curve (CEAC) and the incremental cost-effectiveness plane. At a WTP threshold of 85,698 RMB/QALY (1x per-capita GDP), the probability that combined therapy is cost-effective exceeded 95%, and the combined therapy was dominant in more than 95% of PSA iterations.

**Table 9:** Health economic evaluation of three plans

	Western Medicine	TCM	Combined
Total cost	13,731	12,312	12,760
QALYs	2.71	2.75	2.81
CUR	5,066.79	4,477.09	4,540.93
Incremental analysis			
ΔCost vs. TCM	+ 1,419	-	+ 448
ΔQALY vs. TCM	-0.040	-	+ 0.060
ICUR vs. TCM	Dominated	-	7,467
ΔCost vs. Western	-	1,419	-971
ΔQALY vs. Western	-	+ 0.040	+ 0.100
ICUR vs. Western	-	Dominant	Dominant

### Sensitivity analysis

**Fig. 6:**
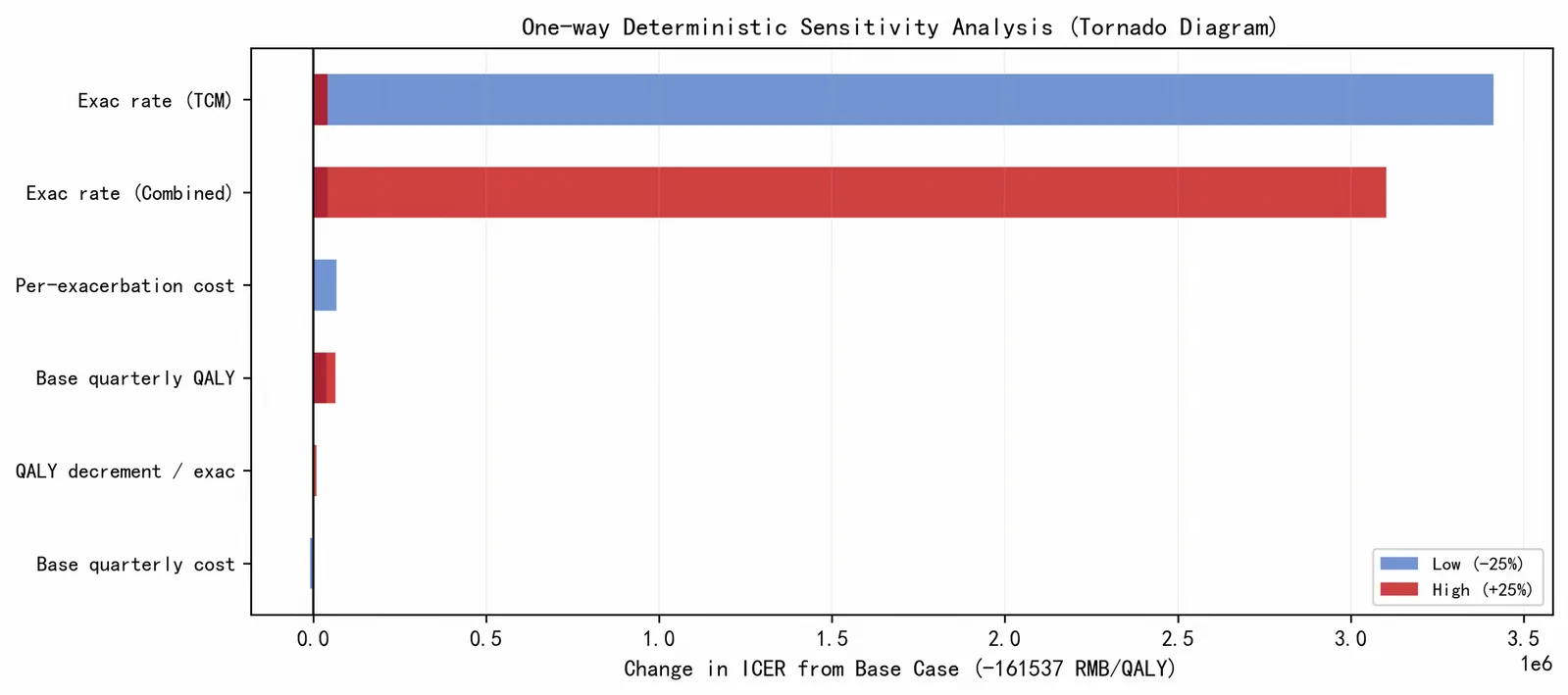
One-way sensitivity analysis (tornado diagram)

To offset potential temporal bias, we conducted probabilistic sensitivity analysis and multi-scenario threshold simulation covering variable ranges of medical costs and COPD acute exacerbation risks, which ensured robust and reliable economic evaluation results. Figure 6 shows the tornado diagram for one-way deterministic sensitivity analysis. Six key parameters were varied by ± 25%; the acute exacerbation cost was the most influential parameter, and none of the perturbations reversed the cost-effectiveness conclusion at the WTP threshold of 85,698 RMB/QALY.

## Discussion

### Key findings

This study developed a discrete event simulation (DES) model to evaluate the cost-utility of Western medicine, Traditional Chinese Medicine (TCM), and combined therapy for stable chronic obstructive pulmonary disease (COPD) from a Chinese healthcare system perspective. Combined therapy achieved the most favorable balance of cost and health outcomes, with an incremental cost-utility ratio (ICUR) substantially below the willingness-to-pay (WTP) threshold of 85,698 RMB/QALY. The 0.060 QALY gain associated with combined therapy corresponds to approximately 22 days in full health and falls within the reported minimum clinically important difference (MCID) range for COPD (0.03–0.07 QALY), suggesting that the benefit may be clinically perceptible at the group level; individual-level variation nonetheless warrants caution.

### Comparison with existing literature

The DES model is still in its infancy in the field of world health economics research, and as of now, there are no relevant empirical studies in China, and there are also very few theoretical research literature. Hoogendoorn [9, 10] established a DES model for COPD to evaluate the incremental costs and effects of different treatment options for various subgroups of COPD patients. Another advantage of this model is that it was constructed using comprehensive data from five large-scale COPD trials [23–27], which can provide some reference and inspiration for this study. Most previously published COPD models are essentially Markov models, which classify the severity of COPD based solely on the degree of airflow obstruction and allocate these health states to COPD deterioration rate, cost, and utility; these models can only report a limited number of results, mainly related to the evaluation of drug treatment plans. Different from these conventional Western medical simulation models [11], this study constructed an innovative, dynamically updated DES model for COPD health economic assessment. This tailored dynamic DES evaluation framework fills the methodological gap in dynamic health economic simulation comparing TCM, Western medicine, and combined interventions for stable COPD. The main structural and methodological differences between the present model and the Hoogendoorn model are summarized in Table 10.

**Table 10:** Differences from hoogendoorn model

Difference	Hoogendoorn model	Topic Model
Data sources	5 large-scale clinical trials	Small multicenter clinical trials
Patient population	Evaluate the efficacy of tiotropium bromide in COPD patients	Patients undergoing stable COPD group therapy
Treatment plan	Thiotropium bromide drug	Three types: Traditional chinese medicine group, Western medicine group, and Combination group
Modeling method	Weibull survival regression model Linear mixed effects model Random intercept linear mixed effects model	Weibull regression model Multivariate logistic mixed effects model Random intercept linear mixed effects model
Ending event	Death	Acute exacerbation
Simulation time	Until death	2 years
Baseline characteristics	Variables related to hormone drug use	Variables related to the number of hospitalizations for acute exacerbation
Intermediate ending	Acute exacerbation, pneumonia	/
Specificity scale	SGRQ	COPD-PRO
Analysis perspective	COPD population treated with specific drugs	COPD population treated in groups
Applicable fields	Clinical event analysis and health economics evaluation of patients using tiotropium bromide medication	Clinical event analysis and health economics evaluation of patients treated with traditional Chinese medicine syndrome differentiation

### Clinical and policy implications

From a TCM perspective, the observed improvements in 6-minute walking distance, symptom scores, and reduced exacerbation rates reflect the effect of strengthening healthy qi and dredging pulmonary qi: TCM interventions not only relieve superficial symptoms (cough, sputum, dyspnea) but also regulate visceral function and enhance general resilience, thereby reducing the frequency and severity of acute attacks. Compared with monotherapy, the integrated approach achieves better utility at relatively lower long-term cost, which aligns with the holistic and simultaneous treatment of principal and subordinate aspects in TCM. As a high-burden chronic disease, COPD brings sustained economic pressure to medical care funds and patients [28–30]. This DES-based cost-utility analysis indicates that the integrated therapy group achieves the best long-term cost-effectiveness among the three treatment regimens. It improves lung function and exercise capacity, relieves respiratory symptoms, and reduces acute exacerbations with reasonable medical costs. These stable advantages verify that the integrated group achieves the optimal balance between clinical efficacy and economic benefits, providing reliable evidence for current COPD health resource allocation and medical insurance decision-making.

### Strengths

The DES model is a modeling method that can represent the interactive relationships between individual behavior, individuals and individuals, individuals and groups, and individuals and the environment, and has good flexibility [31, 32]. The core components of DES include subject, attributes, events, resources, queues, and time [33]. Compared with Markov models, discrete models are an individual simulation that does not fix the time points of events and memorizes various clinical events. DES is more suitable for model construction in situations where resources are constrained or restricted, there are interdependent relationships between individuals or between individuals and the environment, events occur at different times, individual characteristics have a significant impact on the model, and researchers focus on events experienced by individuals [34, 35]. 

Importantly, the core contribution of this study is not dependent on novel raw data, but on the establishment of a TCM intervention-based DES simulation framework for COPD health economic evaluation. Guided by the TCM pathogenesis theory of root deficiency and branch excess, this dynamic modelling paradigm realizes quarterly outcome updating via multilevel mixed-effects models and Weibull regression and reflects the holistic regulatory effects of TCM and integrated TCM-WM treatment. It can serve as a standardized template for future TCM health economic studies with updated prospective data.

### Limitations

Several limitations should be acknowledged. First, the model was parameterized using data from a single clinical trial (328 patients), which limits the generalizability of the findings to broader COPD populations. Second, the regression models were estimated from this single dataset, and there is a risk of overfitting; the external validity of the parameter estimates therefore warrants confirmation in independent cohorts. Third, the trial was conducted in a single region in China, and geographic differences in healthcare delivery, drug pricing, and disease management may limit the transferability of the cost-effectiveness results to other settings. Fourth, some indicators of the model are not optimal choices due to data limitations; for sports activities, the clinical expert group believes that daily steps are the best measure, and this time it is defined by 6MWD. Calculating the utility of QALYs is not a direct measurement, but rather a utility value derived from SF-36. In addition, there is a lack of continuous follow-up data for some other related indicators of lung function. Because the regression models were estimated from data obtained from comparative benefit trials using specific drugs and interventions, the patient characteristics of different interventions vary, limiting the extrapolation of results to the general COPD population. Consequently, the present findings should be regarded as exploratory and hypothesis-generating, requiring validation in larger, geographically diverse multi-center studies.

We also performed an external validation of the uniform approximation used for event-time generation on an independent observational COPD cohort (*N* = 531, quarterly visits, 50 first-exacerbation events). The Δ/median ratio was 0.034 (vs. 0.08–0.125 in the DES trial), confirming that the uniform approximation is a property of the quarterly follow-up design rather than an artifact of the trial sample. However, the Kolmogorov–Smirnov distance was 0.20 in the external cohort (vs. < 0.01 in the DES data), reflecting the different endpoint definition (first vs. recurrent exacerbations). The uniform approximation is most tightly justified for recurrent-event models where the hazard is approximately constant; for first-event analyses, the conditional Weibull draw (see Supplementary Appendix) is recommended as the primary approach. These boundary conditions should be considered when applying the model to settings with different endpoint structures.

### Future directions

COPD has problems such as unclear disease diagnosis and delayed treatment, and most patients have comorbidities; regional differences are also an important influencing factor. Therefore, more representative real-world data of COPD populations can be used to re-estimate models and equations to evaluate whether there are significant differences in the results [36, 37]. Real-world databases usually do not contain as many different parameters as this research model, and they also contain many missing values; choosing the appropriate database is particularly important. Future studies with updated prospective data and dynamic cost modules linked to simulated event frequency and severity are warranted to further validate the model’s generalizability.

## Conclusions

Exploring the disease burden assessment method based on DES model under the intervention of traditional Chinese medicine using COPD as a demonstration, and assisting the development and improvement of health economics technology/methods in the field of traditional chinese medicine. Although the study uses real-world data with certain time limitations, the DES framework with dynamic outcome updating provides a novel and replicable method for evaluating the health economics of TCM. The findings support that TCM-Western integrated therapy, with both symptom control and holistic regulation, is a cost-effective strategy worthy of clinical promotion and policy attention.

## Supplementary Information

Below is the link to the electronic supplementary material.

Supplementary Material 1

## Data Availability

No datasets were generated or analysed during the current study.

## Abbreviations

COPD: Chronic Obstructive Pulmonary Disease
DES: Discrete Event Simulation
TCM: Traditional Chinese Medicine
6MWD: 6-Minute Walking Distance
FEV1: Forced Expiratory Volume in 1 s
QALY: Quality-Adjusted Life Year
CUR: Cost-Utility Ratio
ICUR: Incremental Cost-Utility Ratio
BMI: Body Mass Index
COPD-PRO: COPD Patient Reported Outcome
