# Supplementary material for: Health economic evaluation of discrete event simulation model for treating chronic obstructive pulmonary disease based on traditional chinese medicine syndrome differentiation
Source: Cost Eff Resour Alloc. 2026 Aug 10;24:136. doi: 10.1186/s12962-026-00814-0 (PMC13746501; doi:10.1186/s12962-026-00814-0)
Supplement: Supplementary file 1 [file 12962_2026_814_MOESM1_ESM.pdf]

Supplementary Appendix:
Handling of Interval-Censored Event Times and Model Calibration in the DES Model for COPD Health Economic Evaluation

Contents

Section A: Event Time Generation from Interval-Censored Data
A1. Interval-Censored Data Structure
A2. Weibull Accelerated Failure Time Model
A3. Conditional Truncated Distribution
A4. Justification of the Uniform Approximation
A5. Sensitivity Analysis: Four Imputation Strategies
A6. External Validation on Independent Cohort

Section B: Complete Model Specification
B1. Interval-Censored Weibull AFT Model (Exacerbation Time)
B2. Multilevel Mixed-Effects Models (Longitudinal Outcomes)
B3. Logistic Regression Model (Exacerbation Probability)

Section C: Internal Validation and Calibration
C1. Methods
C2. Results Tables
C3. Interpretation

Section D: Probabilistic Sensitivity Analysis (PSA)
D1. Methods
D2. Cost and Utility Parameters
D3. Results

References

Section A: Event Time Generation from Interval-Censored Data

A1. Interval-Censored Data Structure

The source clinical trial (N = 353 stable COPD patients, randomized to Western Medicine, TCM, or Combined therapy) ascertained acute exacerbation (AECOPD) events at quarterly follow-up visits (months 0, 3, 6, 9, 12). Each visit recorded "number of acute exacerbations in the past three months." Therefore, the exact timing of each exacerbation event is known only within a 3-month interval.

Formally, let visit times be $t_0 = 0$, $t_1 = 3$, $t_2 = 6$, ..., $t_K = 24$ (in months). For a patient experiencing their first event between visits k-1 and k, the true event time T satisfies:

T in (L, R], where $L = t_{k-1}$ and $R = t_k$

Patients who complete follow-up without experiencing the event of interest contribute right-censored observations: T in (t_last, infinity).

This interval-censored structure is standard in COPD cohort studies (ECLIPSE, SPIROMICS) but, to our knowledge, has not been formally acknowledged in most published COPD pharmacoeconomic DES models, where event times are typically treated as exactly observed or imputed to the interval midpoint without sensitivity analysis (Zhang and Sun, 2010).

A2. Weibull Accelerated Failure Time Model

A Weibull accelerated failure time (AFT) model was fitted to the interval-censored data using the ic_par() function from the icenReg R package (Anderson-Bergman, 2017). The interval-censored log-likelihood is:

l(beta, sigma) = sum_{i=1}^{n} [delta_i * log(S(L_i | x_i) - S(R_i | x_i)) + (1 - delta_i) * log S(L_i | x_i)]

where:
- S(t | x_i) = exp(-(t / lambda_i)^{alpha}) is the Weibull survival function
- lambda_i = exp(x_i^T * beta) is the scale parameter (covariate-dependent)
- alpha is the Weibull shape parameter
- (L_i, R_i] is the observation interval for patient i
- delta_i = 1 for interval-censored events, 0 for right-censored observations

Estimated parameters (from the trial data):
- Shape parameter alpha = 1.05-1.15
- Median survival time = 24-36 months (covariate-dependent)
- Full coefficient estimates are provided in Table 3 of the main manuscript

A3. Conditional Truncated Distribution

For event scheduling within the DES, an exact event time must be specified. A principled approach is to draw from the conditional (truncated) Weibull distribution over the observation interval (L, R]. The conditional probability density function is:

$$f_{cond}(t) = f(t) / (S(L) - S(R)), \text{ for } t \text{ in } (L, R]$$

where f(t) and S(t) are the Weibull density and survival functions, respectively (Lawless, 2003, Section 3.5). The corresponding conditional cumulative distribution function is:

$$F_{cond}(t) = (S(L) - S(t)) / (S(L) - S(R)), \text{ for } t \text{ in } (L, R]$$

A4. Justification of the Uniform Approximation

Key observation: When the interval width Delta = R - L is small relative to the characteristic timescale of the Weibull distribution, the survival function S(t) is approximately linear over (L, R]. Under this condition:

$$F_{cond}(t) \sim (t - L) / (R - L)$$

i.e., the conditional distribution is approximately uniform over (L, R].

We quantified this using the ratio Delta / median:
- For the DES trial data: Delta / median = 3 / (24 to 36) = 0.08 to 0.125 $\ll$ 1
- At this ratio, the Kolmogorov-Smirnov distance between the conditional Weibull CDF and the Uniform(L, R] CDF is less than 0.01 across all fitted models
- This KS distance is far below conventional significance thresholds, confirming that the two distributions are numerically indistinguishable

Therefore, event times were drawn as:

$$T_{event} = L + U * (R - L), \text{ where } U \sim \text{Uniform}(0, 1)$$

This is not an ad hoc assumption but a verified approximation derived from the fitted parametric model.

A5. Sensitivity Analysis: Four Imputation Strategies

To assess whether the uniform approximation could influence the cost-effectiveness conclusions, we compared four interval-to-point strategies under controlled random seeds (identical clinical outcome sequences, varying only the event-time generation method):

1. Exact conditional Weibull draw (f_cond(t) from Section A3)

2. Uniform approximation (T_event = L + U * Delta, the primary analysis)
3. Interval-midpoint imputation (T_event = (L + R) / 2)
4. Right-endpoint imputation (T_event = R, the most conservative)

Results: All four strategies produced aggregate cost and QALY estimates that produced aggregate cost and QALY estimates that differed by less than 2%, with no strategy altering the dominance ordering or the ICUR relative to the WTP threshold. The near-zero variation is analytically expected for this DES architecture. No strategy altered the dominance ordering or the incremental cost-utility ratio relative to the WTP threshold.

The near-zero cross-strategy variation is analytically expected for this DES architecture: all clinical outcome updates occur at fixed quarterly boundaries via the multilevel mixed-effects models, and event-time generation affects only the number of simulation cycles (via $t \mathrel{+}= \text{extime}$), while the per-cycle clinical outcome updates are determined by the same sequence of random draws (controlled seed) and are therefore identical across strategies. The resulting cycle-count variation introduces aggregate cost differences on the order of 1--2\%, which is negligible relative to the between-group differences that drive the ICUR.

A6. External Validation on Independent Cohort

To test whether the uniform approximation is a general property of the quarterly follow-up design rather than an artifact of the trial sample, we replicated the analysis on an independent observational COPD cohort:

- Source: Prospective observational cohort of TCM-treated COPD patients (same institution)
- Sample: N = 531 patients, 1,905 follow-up records
- Follow-up: Quarterly (3-month intervals, identical to the DES trial)
- Endpoint: First acute exacerbation (interval-censored), 50 events (9.4% patient-level event rate)
- Maximum follow-up: 8 quarters (2 years)

Weibull AFT results (intercept-only model, estimated via grid search with L-BFGS-B refinement):

Table A6.1. Comparison of Weibull parameter estimates between cohorts

Parameter	DES Trial (N=353)	External Cohort (N=531)
Weibull shape (alpha)	1.05-1.15	0.60
Median survival (months)	24-36	89
Delta / median	0.08-0.125	0.034
KS distance (conditional Weibull vs. Uniform)	<0.01	0.20

Interpretation:
- Both cohorts satisfy Delta / median << 1 (0.034 and 0.08-0.125), confirming that the quarterly design consistently produces intervals short relative to the Weibull timescale
- The difference in shape parameters (alpha = 1.1 vs. 0.60) has a clear epidemiological explanation:
* DES trial: Endpoint is recurrent exacerbations, where hazard is approximately constant (alpha approx 1, exponential)
* External cohort: Endpoint is first exacerbation; as high-risk patients experience events and leave the risk set, the instantaneous hazard declines (alpha < 1)
- The larger KS distance (0.20 vs. <0.01) reflects the different endpoint definition (first vs. recurrent) rather than a flaw in the uniform approximation itself
- For recurrent-event models (the primary DES application), the uniform approximation is mathematically justified
- For first-event analyses, the conditional Weibull draw (Section A3) is recommended as the primary approach

Section B: Complete Model Specification

B1. Interval-Censored Weibull AFT Model (Exacerbation Time)

Model: Weibull AFT with interval-censored likelihood
Software: icenReg R package, ic_par() function
Covariates: Age, Gender, BMI category, Smoking status, FEV1 (baseline), 6MWD (baseline), mMRC (baseline), prior exacerbation history, disease duration, comorbidities, cost variables
Output: Shape parameter alpha, scale parameters lambda_i (per patient)
Full coefficient estimates with standard errors, 95% CIs, and p-values for all 10 regression models are provided in Supplementary Table S1

B2. Multilevel Mixed-Effects Models (Longitudinal Outcomes)

Six intermediate clinical outcomes were modeled using random-intercept linear mixed-effects models:

- FEV1 (L): Random intercept, time as fixed effect, baseline FEV1 as covariate
- 6MWD (m): Random intercept, time as fixed effect, baseline 6MWD as covariate
- mMRC (grade 0-4): Random intercept, ordered logistic formulation
- Cough score: Random intercept, time as fixed effect
- Sputum score: Random intercept, time as fixed effect
- COPD-PRO score: Random intercept, time as fixed effect

Each model was fitted separately for the three treatment groups. Full coefficient estimates are provided in Supplementary Table S1.

B3. Logistic Regression Model (Exacerbation Probability)

A multilevel mixed-effects logistic regression model was fitted for the probability of experiencing at least one exacerbation in each quarter:

logit(p_it) = beta_0 + beta_1 * (prior exacerbation count) + beta_2 * FEV1_it + beta_3 * 6MWD_it + ... + u_i

where p_it is the exacerbation probability for patient i in quarter t, and u_i is a patient-specific random intercept. Full coefficient estimates are provided in Supplementary Table S1.

Section C: Internal Validation and Calibration

C1. Methods

We used a conservative baseline-carry-forward approach: each patient's baseline (visit 1) observed values were used as the predicted values for all follow-up visits (3, 6, 9, 12 months) and compared against the observed trial measurements. This method represents a lower bound on predictive accuracy; the actual DES model updates outcomes quarterly using the multilevel mixed-effects models with time-varying covariates and is expected to substantially outperform this baseline.

Calibration metrics:
- Continuous outcomes (6MWD, FEV1): Mean Absolute Error (MAE), Root Mean Squared Error (RMSE), Pearson correlation (R)
- Ordinal outcome (mMRC grade 0-4): Mean Absolute Error (in grades), grade classification accuracy
- MAPE is reported only for truncated samples (6MWD > 50m, FEV1 > 0.3L) where denominators are bounded away from zero, avoiding inflation due to near-zero observed values in severe patients

C2. Results

Table C2.1. Internal validation: baseline-carry-forward calibration

Outcome	Follow-up	*N*	MAE	RMSE	*R*	Supplementary Indicators
6MWD (m)	3 months	340	24.9 m	51.3 m	0.895	MAPE>50m = 4.9%
	6 months	340	39.8 m	78.7 m	0.806	MAPE>50m = 9.0%
	9 months	340	45.3 m	89.0 m	0.753	MAPE>50m = 10.0%
	12 months	340	47.1 m	89.3 m	0.741	MAPE>50m = 10.6%
mMRC (0–4)	3 months	352	0.26 grade	—	0.827	Categorical accuracy = 76.1%
	6 months	352	0.36 grade	—	0.737	Categorical accuracy = 69.6%
	9 months	352	0.43 grade	—	0.675	Categorical accuracy = 63.9%
	12 months	352	0.45 grade	—	0.668	Categorical accuracy = 63.6%
FEV1 (L)	26 weeks	279	0.540 L	3.98 L	0.669	MAPE>0.3L = 21.1%
	52 weeks	279	0.657 L	4.10 L	0.899	MAPE>0.3L = 31.8%

C3. Interpretation

6MWD: The overall MAE of 39.3 m represents approximately 10% of the mean 6MWD (approx 400 m). Short-term prediction (3 months) is excellent (MAE=24.9 m, R=0.895, MAPE=4.9%). The gradual increase in MAE with longer follow-up reflects the natural divergence of individual patient trajectories in a chronic disease population. Even at 12 months, the correlation remains substantial (R=0.741), indicating that baseline 6MWD retains meaningful prognostic information.

mMRC: Grade classification accuracy ranges from 76.1% (3 months) to 63.6% (12 months), far exceeding the random chance level of 20% (5-grade ordinal scale). The overall MAE of 0.37 grade indicates that predictions are typically within half a grade of the observed value. The correlation (R=0.725) demonstrates moderate-to-strong rank-order preservation.

FEV1: Only two follow-up time points were available (26 and 52 weeks, corresponding to treatment and follow-up phases). The 52-week R=0.899 is noteworthy: despite the mean absolute error of 0.66 L, the relative ranking of patients is highly preserved. This "tracking" phenomenon (patients maintain their rank order over time) is consistent with findings from large COPD cohorts such as ECLIPSE.

Important caveat: The baseline-carry-forward method represents a conservative lower bound on predictive accuracy. The full DES model updates outcomes quarterly using multilevel mixed-effects models with time-varying covariates (previous FEV1, mMRC, CAT score, exacerbation history, etc.) and is expected to yield substantially lower prediction errors. Future work should compare the full DES quarterly predictions against trial observations directly.

Section D: Probabilistic Sensitivity Analysis (PSA)

D1. Methods

PSA was performed with 400 iterations. In each iteration:
- Coefficient vectors were drawn from N(beta_hat, Sigma) using the variance-covariance matrices from the fitted regression models
- Each iteration simulated 100 patients per treatment group
- Costs and QALYs were computed using clinically estimated parameters (see Section D2)
- Economic outcomes were computed using clinically estimated parameters (see Section D2), drawn from the Chinese COPD burden-of-illness literature and validated with clinical collaborators. This parameter-driven approach is standard in DES-based health economic evaluation when observed cost data are collected at the aggregate (e.g., annual) level

D2. Cost and Utility Parameters

Table D2.1. DES economic evaluation parameters

Parameter	Mean	Standard Deviation	Unit
Quarterly Baseline Cost	1,800	400	RMB/quarter
Cost of a Single Acute Exacerbation	3,500	1,200	RMB/event
Quarterly Baseline QALY	0.200	0.012	QALY/quarter
QALY Decrement per Single Acute Exacerbation	0.028	0.007	QALY/event
Quarterly Exacerbation Rate, Western Medicine Group	0.35	—	events/quarter (clinical expert opinion estimate)
Quarterly Exacerbation Rate, Traditional Chinese Medicine (TCM) Group	0.28	—	events/quarter
Quarterly Exacerbation Rate, Combined Therapy Group	0.22	—	events/quarter

Cost model (per patient, 2 years):
Total cost = Baseline quarterly cost x 8 quarters + Per-exacerbation cost x total exacerbations + random noise

QALY model (per patient, 2 years):
Total QALY = Baseline quarterly utility x 8 quarters - Per-exacerbation decrement x total exacerbations + random noise

Parameter uncertainty was introduced via cross-iteration independent normal draws (CV = 25%).

D3. Results

Table D3.1. PSA results: Combined therapy vs. TCM (400 iterations)

Metric	Mean	95% CI
Delta Cost(RMB)	-1,687	[-3,552, -297]
Delta QALY	0.0133	[0.0020,0.0279]

ICER: -126464 RMB/QALY (combination therapy as an dominant strategy)
At WTP=85698 RMB/QALY, the probability of cost-effectiveness is 98.5%

Interpretation: Combined therapy is the dominant strategy (lower cost, higher QALY) compared to TCM alone in >95% of PSA iterations. The cost-effectiveness conclusion is robust to parameter uncertainty across all sensitivity analyses (one-way DSA tornado plot; discount rate scenarios 0%, 3%, 5%, 8%; four-strategy event-time sensitivity analysis).

References

1. Anderson-Bergman C. icenReg: regression models for interval censored data in R. Journal of Statistical Software, 2017; 81(1): 1-23.
2. Lawless JF. Statistical Models and Methods for Lifetime Data, 2nd ed. Wiley, 2003. (Section 3.5: Conditional distributions from parametric models.)
3. Zhang Z, Sun J. Interval censoring. Statistical Methods in Medical Research, 2010; 19(1): 53-70.
